# Art Produced By a Patient with Parkinson’s Disease

**DOI:** 10.1155/2006/901832

**Published:** 2006-07-17

**Authors:** Anjan Chatterjee, Roy H. Hamilton, Prin X. Amorapanth

**Affiliations:** Department of NeurologyUniversity of Pennsylvania School of MedicinePhiladelphiaPA 19104USA

**Keywords:** Aesthetics, neuro-aesthetics, movement disorders

## Abstract

Artists with neurological diseases sometimes produce remarkably appealing art. Here we present the art of a patient with Parkinson’s disease, who demonstrated remarkable creativity and productivity well into the course of his disease. Despite his manifest motor impairments, he produced paintings and drawings with fluid sinuous movements. He also became preoccupied with his art in an unprecedented way, raising the possibility that his disease and medications were contributing to his creative drive.

Brain damage can impair our ability to speak or comprehend language, to coordinate movements, to recognize objects, to apprehend emotions and to make logical decisions. In contrast to these cognitive abilities, brain damage sometimes paradoxically improves artistic production. Artistic changes have been described in stroke, migraine, epilepsy, autism, Alzheimer's disease and fronto-temporal dementias (FTD) [1,2]. One patient with PD has been described in whom poetic talents were uncovered by treatment [3]. Relatively little is known about visual art in Parkinson's disease (PD), even though public shows occasionally display such art [4,5]. Here, we describe a patient with PD, who became artistically productive well into the course of his disease. His artistic output offers insight into the relationship of motor control in art and PD. It also raises questions about the neural underpinnings of the creative drive.

**Case report.** CSD was a 68-year-old right-handed graphic designer with a 15-year history of PD. His initial symptoms were tremor and rigidity in the right arm and in 1992 he was started on Sinemet 10/100 four times daily and Selegiline 5 mg twice daily. After progression of right-sided symptoms, in 1997 his medications were increased to Sinemet CR 25/100 every 5 hours with additional doses of Sinemet 25/100 as needed. Pergolide, Ropinerol, and Pramipexole were not tolerated. In late 2002 his motor function declined more precipitously. He stopped taking Selegiline and started taking Entacapone and then Amantadine. He was restarted on Pramipexole in March 2003, which he tolerated. He experienced occasional attacks of sleepiness and motor fluctuations, but no dyskinesias. His medications in 2005 were Sinemet 25/100 every 3 hours, Entacapone 200 mg every 3 hours from 6 am to 9 pm, Amantadine 100 mg three times daily, and Pramipexole 0.5 mg three times daily. He also took Tamsulosin 0.4 mg daily and Atorvastatin 10 mg daily.

CSD was alert and oriented without evidence of dementia. His language was fluent and he comprehended well. He had a masked face, a resting tremor in the right arm and left leg. His movements were bradykinetic bilaterally, most prominently in the right arm. He had cogwheel rigidity in both arms. His writing was micrographic and tremulous (Fig. 1). He could arise from a chair with some difficulty. He could accomplish activities of daily living slowly but without assistance.

ISSN 0953-4180/06/$17.00 © 2006 – IOS Press and the authors. All rights reserved

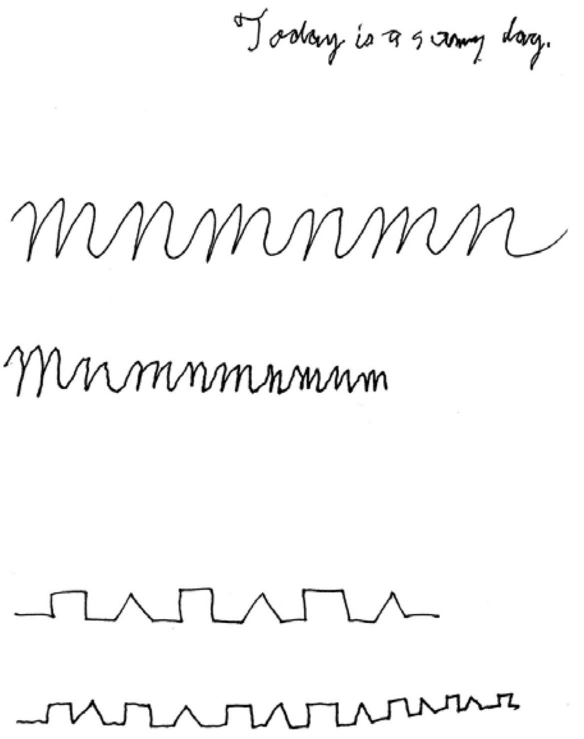

Fig. 1. CSD's writing demonstrating his micrographia and tremulousness.

He was Hoehn and Yahr stage 2, defined by bilateral disease without impairment of balance.

**CSD's art.** With the encouragement of a psychologist that CSD saw in November of 2001 for depression, he began to paint and draw. He draws with his right hand despite its tremor, rigidity, and bradykinesia. Some of CSD's initial art was representational, inspired by painters such as Van Gogh. These works often depicted landscapes (Fig. 2a). They are approximately 2' $\times$ 3'in physical size and employ a full range of vivid colors. The lines in these early works are regular in thickness and spacing, qualities retained in CSD's subsequent work. A year later, CSD began to use colored pencil, which allowed him to produce finer lines. His compositions became more abstract (Fig. 2b). He drew without the assistance of French curves and his lines were regular and even. Some of CSD's work displayed a restricted color palette of one or two hues, and an overall shift towards a denser and darker tonality. Other pieces were colored more vibrantly. His art continued to be relatively abstract. He lives in an apartment overlooking a city park with a square configuration. His more recent work developed a visual theme with a central square inspired by this view (Fig. 2c).

CSD describes himself as being obsessed by his art. When he draws, he has "a sense of bursting forth and tearing back walls." He repeats variations on specific themes and has produced hundreds of works in the last several years. He feels a strong urgency to produce, which he describes as follows: "The train has left the station and I have just been served a delicious dinner in the café car. The train is picking up speed so I have to eat fast so I can finish my meal before we get to the last stop and I have to get off." As an art student in his youth, and as an adult who painted sporadically, he never felt his recent sense of urgency. He notes that his movements are in complete control when drawing, despite being frustratingly impaired in other contexts. He thinks it possible that his disease and medications are contributing to his artistic generativity.

## 1. Discussion

CSD was remarkably productive artistically well into the course of his disease. He felt an urgent need to express himself and wondered about the role of PD in his artistic production. His experiences are similar to those of Johanne Vermette, an artist and physician with PD, who thought her paintings were enhanced since her diagnosis [5]. She said that her paintings were "less precise but more vibrant" and "I have a need to express myself more. I let myself go, sometimes painting with enraged fingers." She also wondered about the role of her disease in her enhanced imagination.

Lakke [4] observed that some PD artists continue to create despite progression of their disease. They often use a cross hatching style that makes use of their tremor. CSD was bradykinetic, rigid, had a resting tremor and his writing was micrographic. By contrast, his artistic movements were fluid. He did use the kind of cross-hatching style (Fig. 2b) described by Lakke, but his dominant use of line demonstrated exquisite control over larger amplitude sinuous movements that did not change directions suddenly. Current models of motor control distinguish distal (used in writing and grasping) from proximal (used in reaching) systems, which can be impaired selectively [6]. CSD's graphic impairment was most evident with distal movements. The specific artistic style he adopted emphasized larger amplitude proximal movements that were relatively preserved.

CSD was preoccupied with his art. His disturbed sleep patterns meant that he awoke early and began painting. His complete immersion into a visual image precluded working on more than one painting at a time. He worked through variations of visual themes, and had an impressive output over a relatively short time.

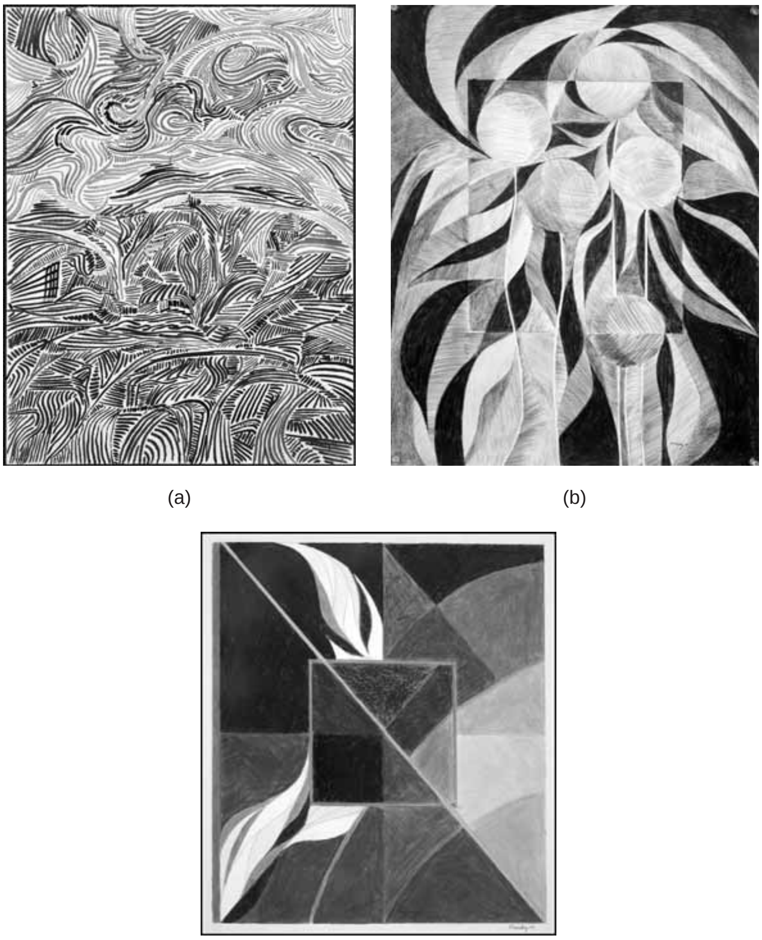

(c)

Fig. 2. (a) Landscape inspired by Van Gogh (original in color); (b) Abstraction showing a combination of the cross hatching style as well as the control over larger amplitude sinuous lines (original in color); (c) Abstraction inspired by the view of the city park from his apartment (original in color).

Obsessive-compulsive traits produced by neurologic disease can create a disposition to produce art, as seen in FTD and autism. FTD artists can work compulsively on visual themes [7]. Artistic children with autism can become preoccupied with drawing specific objects, such as horses or buildings [8]. Obsessive-compulsive symptoms are mediated by dopaminergic and serotonergic systems [9]. Some PD patients on dopamine agonists develop dysfunctional obsessive-compulsive behaviors such as uncontrolled gambling [10]. CSD may have had such personality traits exaggerated by his medications, expressed not in gambling, but in visual art.

One is reluctant to reduce CSD's creativity to the ravages of PD. However, his experience points out that a subtle interplay of motor control, visual imagination and drive contributes to artistic production. The modulation of these factors by disease and medications seems to have cleared a conduit for his creative expression.

## Acknowledgment

We would like to thank CSD for his permission to publish examples of his images.
